# Sample Treatment with Trypsin for RT-LAMP COVID-19 Diagnosis

**DOI:** 10.3390/biology12070900

**Published:** 2023-06-23

**Authors:** Soraya García-Sorribes, Francisco Lara-Hernández, Iris Manzano-Blasco, Jessica Abadía-Otero, Eliseo Albert, Alba Mulet, Laisa Socorro Briongos-Figuero, Miriam Gabella-Martín, Ignacio Torres, Jaime Signes-Costa, David Navarro, Juan-Carlos Martín-Escudero, Ana-Bárbara García-García, Felipe Javier Chaves

**Affiliations:** 1 Genomic and Diabetes Unit, INCLIVA Biomedical Research Institute, 46010 Valencia, Spain; 2 Internal Medicine Service, Rio Hortega University Hospital, 47012 Valladolid, Spain; 3 Microbiology Service, University Clinic Hospital, INCLIVA, 46010 Valencia, Spain; 4 Pulmonary Department, University Clinic Hospital, INCLIVA, 46010 Valencia, Spain; 5 Medicine Department, Valladolid University, 47002 Valladolid, Spain; 6 CIBERDEM, ISCIII, 28029 Madrid, Spain

**Keywords:** COVID-19, SARS-CoV-2, RT-LAMP, RNA extraction-free, trypsin, colorimetry

## Abstract

**Simple Summary:**

At the end of 2019, a new virus named SARS-CoV-2, which causes COVID-19 disease, emerged. It was recognized as a pandemic on 11 March 2020 and caused the collapse of hospital systems in several countries due to the overflow of patients. In this context, several detection techniques have been developed, among them, the RT-qPCR achieve the best sensitivity and specificity for virus detection although it is expensive and time-consuming and needs a previous RNA sample extraction. Therefore, the need to speed up the process has led to the development of rapid tests such as antibody-antigen tests, which allow diagnosis in less time, although they have lower sensitivity and specificity. Therefore, we propose a test for SARS-CoV-2 diagnosis that is faster than RT-qPCR and has higher sensitivity and specificity than antigen tests. This test is based on the use of trypsin for sample treatment without an RNA extraction step and the subsequent amplification by Colorimetric Reverse Transcription Loop-Mediated Isothermal Amplification. This point-of-care procedure allows COVID-19 diagnosis in 40 min with colorimetric detection in no specialized centers and could be applied to other viruses.

**Abstract:**

The SARS-CoV-2 coronavirus is responsible for the COVID-19 pandemic resulting in a global health emergency. Given its rapid spread and high number of infected individuals, a diagnostic tool for a rapid, simple, and cost-effective detection was essential. In this work, we developed a COVID-19 diagnostic test, that incorporates a human internal control, based on the Reverse Transcription Loop-Mediated Isothermal Amplification (RT-LAMP). When working with synthetic SARS-CoV-2 RNA, the optimized RT-LAMP assay has a sensitivity of 10 viral copies and can be detected by fluorescence in less than 15 min or by the naked eye in 25 min using colorimetric RT-LAMP. To avoid the RNA extraction step, a pre-treatment of the sample was optimized. Subsequently, a validation was performed on 268 trypsin treated samples (including nasopharyngeal, buccal, and nasal exudates) and amplified with colorimetric RT-LAMP to evaluate its sensitivity and specificity in comparison with RT-qPCR of extracted samples. The validation results showed a sensitivity and specificity of 100% for samples with Ct ≤ 30. The rapid, simple, and inexpensive RT-LAMP SARS-CoV-2 extraction-free procedure developed may be an alternative test that could be applied for the detection of SARS-CoV-2 or adapted to detect other viruses present in saliva or nasopharyngeal samples with higher sensitivity and specificity of the antibody test.

## 1. Introduction

The human coronavirus severe acute respiratory syndrome coronavirus-2 (SARS-CoV-2) was identified in January 2020 and triggered a global public health emergency [1]. The disease caused by SARS-CoV-2 has been named coronavirus disease 2019 (COVID-19), whose carriers may be asymptomatic or present a wide range of symptoms including cough, sputum production, fatigue, headache, myalgia, fever, loss of taste and/or smell, sore throat, and, in severe cases, death [2].

The first cases of COVID-19 were reported in Wuhan on December 2019 and it quickly spread to the rest of the world [1]. On 20 February 2023, a total of 6,844,267 deaths had been recorded worldwide, and currently, the number of new cases in the last 7 days is approximately 824,639 [3]. The pandemic began with an exponential number of deaths per day, which accelerated the emergence of diagnostic techniques to isolate positives and minimize the infection rate [4], the gold standard of which is quantitative reverse transcription PCR (RT-qPCR). It allows the identification and quantification of viral RNA with high sensitivity and specificity after RNA extraction [5]. However, this technique has some limitations: it requires specialized and expensive laboratory equipment, qualified staff, and it is time-consuming [6]. Alternatively, many other tests, such as antibody and antigen tests, have been developed to provide a simple and rapid diagnosis. These immunoassay tests are based on protein-to-protein interaction, which is less specific than molecular assays. Furthermore, they require a high viral load to detect SARS-CoV-2, which varies according to the period of infection. For these reasons, the false negative rate is higher than with RT-qPCR, although these tests are cheaper and faster [7,8,9].

Given the limitations of these diagnostic techniques, many laboratories have worked to develop a test that combines the sensitivity and specificity of RT-qPCR with the advantages of immunological testing [10,11]. An alternative for this purpose is Reverse-Transcription Loop-Mediated Isothermal Amplification (RT-LAMP). This method was developed by Notomi and colleagues and allows isothermal amplification of RNA at 60–70 °C, thus eliminating the need for sophisticated equipment [12,13]. The presence of six target sequences makes the procedure highly specific and generates a loop structure that allows a rapid amplification of the target region in 30–60 min [14]. In addition, results can be determined directly from colour changes in the reaction mix, among other detection systems [15]. RT-LAMP has already been used as a test to detect several pathogens in extracted samples [16,17], including SARS-CoV-2 [18,19].

In this work, we have optimized an RT-LAMP reaction that allows rapid detection of SARS-CoV-2 virus in extracted samples with high sensitivity and specificity. Furthermore, in order to make the test as simple and rapid as possible, we have used a novel sample treatment with trypsin that avoids the RNA-extraction step. In summary, we describe a rapid and simple test that could facilitate the diagnosis of COVID-19.

## 2. Materials and Methods

### 2.1. Standard and Clinical Samples

Standard controls for the test included a synthetic SARS-CoV-2 RNA control (Twist Bioscience, San Francisco, CA, USA) for virus and human RNA extracted from peripheral blood as a human internal control.

The study included 169 nasopharyngeal, 57 nasal exudate, and 42 buccal swab samples collected in saline buffer from the Hospital Clínico Universitario de Valencia (Valencia, Spain), taken between November and December 2020, Hospital Universitario Río Hortega (Valladolid, Spain), taken on December 2020, and Hospital Puerta del Hierro Biobank (Madrid, Spain), which arrived frozen at our facilities on August 2020. During this process, samples were frozen at −80 °C and thawed on ice according to the need for availability for testing.

### 2.2. RNA Extraction

RNA was extracted using the Quick-RNA ViralTM 96 Kit (Zymo Research, Irvine, CA, USA) according to the manufacturer’s instructions, with an initial sample volume of 100 µL and an elution volume of 15 µL in nuclease-free water.

### 2.3. Sample Treatment

After different conditions of trypsin treatment were tested (Table S1), for direct RT-LAMP, samples were incubated in a 2/3 ratio with 0.05% trypsin-EDTA (Gibco, Rodano, Italy) for 5 min at room temperature and then at 95 °C for 5 min.

### 2.4. RT-qPCR

Primers and TaqMan probes were designed using Primer3 software [20] and were purchased from Merck (KGaA, Darmstadt, Germany) and Condalab (Torrejón de Ardoz, Madrid, Spain), respectively. They include the N and RdRP genes as viral targets (NC_045512.2) and the LYSOZYME gene as an internal control (NM_000239.3), sequences shown in Table 1. Each RT-qPCR reaction mix using the Luna^®^ Universal Probe One-Step RT-qPCR Kit (New England BioLabs, Beverly, Massachusetts, UK) contained 1× Luna Probe One-Step Reaction Mix (No ROX), 1× Luna WarmStart^®^ RT Enzyme Mix, 1000 nM of each primer, 250 nM of each TaqMan probe, 3 mM MgCl_2_, and 1 µL of purified sample in a final volume of 15 µL. Reactions were performed on a LightCycler 480 II (Roche, Basel, Switzerland) with a temperature profile of 10 min at 55 °C for reverse transcription, 1 min at 95 °C for initial activation, followed by 45 cycles of 10 s at 95 °C and 30 s at 60 °C for amplification.

### 2.5. RT-LAMP

Primers for the N region of SARS-CoV-2 (NC_045512.2) and the housekeeping human GAPDH gene (NM_002046) as an internal control were designed using PrimerExplorer V5 (http://primerexplorer.jp/lampv5e/, accessed on 23 April 2020) and Primer3 software [20] and were purchased from Merck (KgaA, Darmstadt, Germany) (Table 2). A set of six oligos was designed and pooled for each target. 10× SARS-CoV-2 primer mix was performed by adding 2 µM F3/B3 primers, 4 µM LF/LB primers and 16 µM FIP/BIP primers. A 10× GAPDH primer mix was performed by adding 2 µM F3/B3 primers, 4 µM LF/LB primers, and 8 µM FIP/BIP primers.

Two detection systems were used to analyze the RT-LAMP results. The fluorescent RT-LAMP reaction was performed using the WarmStart LAMP kit (New England BioLabs, Beverly, Massachusetts, UK) according to the manufacturers’ recommendations, 3 mg/mL of Bovine Serum Albumin (BSA, Merck, Darmstadt, Germany), 1× primer mix, and 2 µL of template. The reaction was supplemented to 15 µL with nuclease-free water and amplified for 30 min at 65 °C using the LightCycler 480 II system (Roche, Basel, Switzerland). For the colorimetric system, 2 µL of template was added to 1× WarmStart Colorimetric LAMP Master Mix (New England BioLabs, Beverly, Massachusetts, UK), 3 mg/mL of Bovine Serum Albumin (BSA, Merck, Darmstadt, Germany), 0.1 mM of Tris-HCl pH 8.0 (ABgene/Thermo Fisher Scientific, Epsom, UK), and 1× primer mix. The reaction was supplemented to 15 µL with nuclease-free water and incubated for 30 min at 65 °C in Bio TBD-100 Dry Block Thermostat (Biosan, Riga, Latvia).

### 2.6. Sequencing

To verify the specificity of the primers for the N region of SARS-CoV-2, a library of the RT-LAMP amplification product was prepared using the Nextera XT DNA Library Preparation Kit (Illumina, San Diego, CA, USA) and sequenced using the Miseq^®^ system (Illumina, San Diego, CA, USA). The analysis was performed using the SARS-CoV-2 reference sequence NC_045512.2.

### 2.7. Conservation

Possible scenarios in the developed protocol that could affect virus detection were tested. To verify that the samples could be stored in trypsin solution for at least 17 days at different storage temperatures, 600 µL of three SARS-CoV-2 positive samples, previously tested by RT-qPCR, were taken and treated with 300 µL of trypsin. Three aliquots of 300 µL were then taken from each sample and stored at room temperature, 4 °C and −20 °C, respectively. RT-qPCR and colorimetric RT-LAMP were performed on each sample after 0, 1 and 17 days.

To determine the shelf life of the colorimetric RT-LAMP mix, it was prepared for eight reactions and stored at −20 °C for 15 days. A reaction was then performed by adding 1000, 100, and 10 copies of synthetic SARS-CoV-2 to the mix.

### 2.8. Statistical Analysis

Data were initially analyzed using Microsoft Excel (version 2303). Graphs were generated using Python3 (version 3.6) and R ggplot2 package (version 3.4.2).

Sensitivity and specificity are defined as the accuracy of a test to detect a positive or negative result according to the reference technique, respectively. These parameters were calculated using RT-qPCR results as the reference. 95% CI was calculated using the proportion test performed with the R Stats package (version 4.2.0).

## 3. Results

### 3.1. Sensitivity and Specificity RT-LAMP Assay using Synthetic SARS-CoV-2 RNA

**Figure 1:**
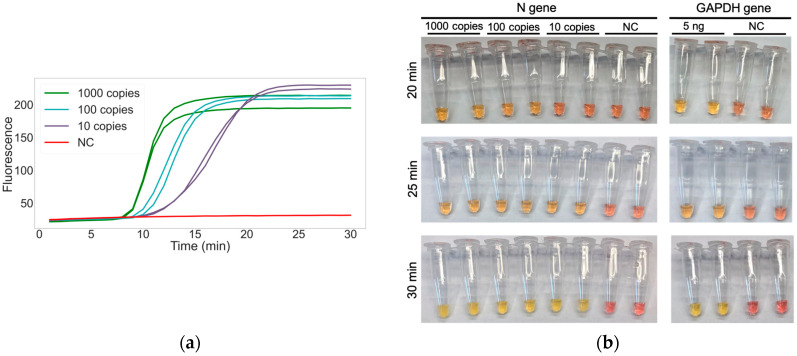
RT-LAMP sensitivity: (**a**) Amplification of two replicates of synthetic SARS-CoV-2 at 1000, 100, and 10 copies by fluorescent RT-LAMP; (**b**) Colorimetric RT-LAMP detection of 1000, 100 and 10 copies of synthetic SARS-CoV-2 (N gene) and 5 ng of human-extracted RNA (GAPDH gene) at 20, 25, and 30 min. NC: negative control, performed with water.

Sensitivity for virus detection was assessed using synthetic SARS-CoV-2 RNA control. By performing serial 10-fold dilutions, a sensitivity limit of 10 copies per reaction was achieved using fluorescent RT-LAMP. Detection times for 1000, 100, and 10 copies were 8.56, 9.65, and 11.40 min, respectively (Figure 1a). The limit detection obtained with colorimetric RT-LAMP protocol was the same as with fluorescent RT-LAMP, although the detection times were slightly longer. After 20 min, a color change was observed in the 1000 and 100 copies in the SARS-CoV-2 synthetic control. After 25 min, the color change was also observed in the 10-copies dilution. A reaction to detect GAPDH as a sample collection control was included in the procedure to determine false negatives (Figure 1b). Serial dilutions of purified total human RNA were performed, and positive amplification was obtained at 0.1 ng.

To ensure the specificity of the amplification, RT-LAMP fragments obtained from a SARS-CoV-2 positive nasal swab sample were sequenced. The results showed that 98.67% of the reads obtained (62,341 out of 63,179 reads) mapped to the N region of the SARS-CoV-2 reference genome.

### 3.2. Sample Treatment and Validation on Clinical Samples with RT-LAMP Assay

Among different proteases, trypsin was selected for sample treatment because of its protein digestion properties in order to eliminate RNAses and inhibitory proteins to the medium. The processed samples were tested by colorimetric and fluorescent RT-LAMP.

A test with selected trypsin treatment on 15 samples using the fluorescent RT-LAMP detection method was performed and it was observed that above 100 copies of SARS-CoV-2 (Ct RT-qPCR ≤ 30) a positive result was obtained in less than 13 min (Figure S1). It was subsequently confirmed that trypsin did not alter the color of the colorimetric RT-LAMP reaction, so we proceeded to the validation of this method.

**Figure 2:**
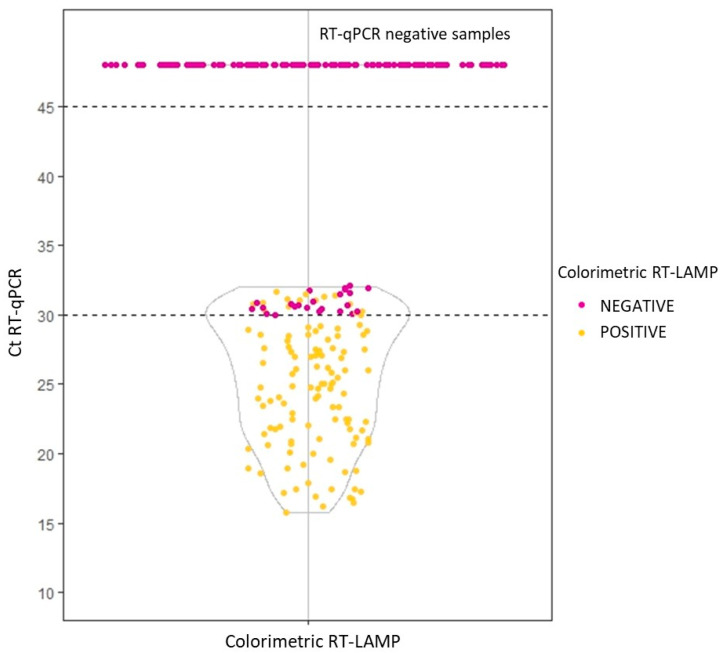
Comparison of colorimetric SARS-CoV-2 RT-LAMP validation results using trypsin versus RT-qPCR of extracted samples.

Validation of this method was performed by colorimetric RT-LAMP employing a total of 268 samples, including nasopharyngeal samples, nasal exudates, and buccal swabs, and compared by RT-qPCR of extracted samples. SARS-CoV-2 RT-qPCR positive samples were divided in two groups based on WHO criteria for an acceptable point-of-care testing [21]: Ct ≤ 30 and Ct > 30 (Ct between 25 and 30, the viral load was around 1000 copies/µL). In the RT-qPCR test, which we compared with RT-LAMP developed test, Ct = 30 corresponds to 100 copies/µL. The sensitivity achieved by colorimetric RT-LAMP in 100 samples with Ct ≤ 30 was 100%, while it decreased to 39.47% (95% CI 31.31% to 63.95%, n = 38) when the Ct > 30. The obtained specificity was 100% (Figure 2). All of them amplified the internal human control.

### 3.3. Evaluation of Sample Conservation in Trypsin Solution and Maintenance of Colorimetric RT-LAMP Reaction Mix

**Figure 3:**
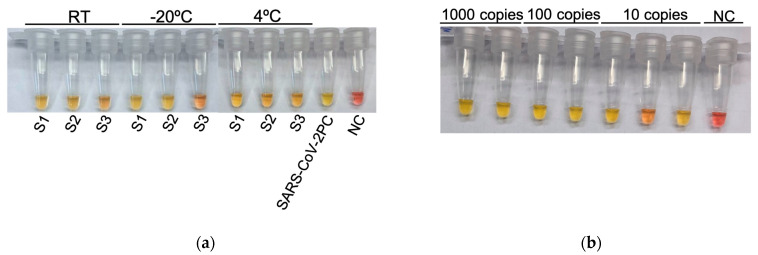
Maintenance of samples and the colorimetric RT-LAMP reaction mix: (**a**) Colorimetric RT-LAMP results on the 17th day of maintenance of three SARS-CoV-2 positive samples in trypsin solution (S1, S2, S3) at room temperature (RT), −20 °C and 4 °C; (**b**) Colorimetric RT-LAMP results after 15 days of maintenance at −20 °C of the prepared colorimetric RT-LAMP reaction mix using 1000, 100, and 10 copies of synthetic SARS-CoV-2. NC: negative PCR control performed with water. SARS-CoV-2 PC: 1000 copies of synthetic SARS-CoV-2 RNA.

To evaluate sample preservation in trypsin solution, three nasal exudate SARS-CoV-2 positive samples previously tested by RT-qPCR were stored at room temperature, 4 °C and −20 °C and tested by colorimetric RT-LAMP. After 17 days, all of them were positive regardless of the storage temperature (Figure 3a). In the same way, to check the maintenance of the colorimetric RT-LAMP reaction mix, it was frozen at −20 °C for 15 days and tested with synthetic SARS-CoV-2 at 1000, 100, and 10 copies and maintained its efficacy (Figure 3b).

## 4. Discussion

With the aim of developing a simple, rapid, and sensitive diagnostic test for COVID-19, in this work we evaluated the use of RT-LAMP assay together with trypsin treatment of samples due to its availability in the laboratory, ease of use, and inactivation with heat, which avoids RNA extraction, compared to conventional RT-qPCR with extracted samples.

Currently, there are several methods for detecting the RT-LAMP product, of which the fluorescent and colorimetric methods were chosen for their speed and simplicity [22]. Our optimized fluorescent RT-LAMP reaction was able to detect up to 10 copies of synthetic SARS-CoV-2 in just 15 min, while with the colorimetric RT-LAMP, the detection time increased to 25 min. This difference could be due to the detection method: fluorescence uses of an intercalating agent instead of a pH indicator [19,20,23]. These detection techniques are useful for different applications. While the fluorescent RT-LAMP method requires specific equipment and must be used in specialized laboratory settings for routine diagnosis, the colorimetric RT-LAMP method can be used in non-specialized settings, as it only requires a constant temperature reaction, and the interpretation of the results can be performed by the naked eye.

In an attempt to avoid the RNA extraction step, we have tested trypsin buffer and heating, which allows the lysis of cells and viruses to release their genetic material into the medium, and the elimination of inhibitors present in samples. We performed a validation using a colorimetric RT-LAMP reaction on 268 clinical samples treated with trypsin. Sensitivity and specificity of 100% were obtained when Ct ≤ 30 (quantified by RT-qPCR). However, the sensitivity decreased when the viral load was low (Ct > 30). In this regard, we observed that extracted samples analyzed by RT-qPCR with a Ct > 30 showed a loss of intensity in the curves, suggesting that in these samples the detection may be affected by the presence of inhibitors [24]. In addition, the state of the viral genome must also be considered, as a high level of fragmentation could negatively affect primer binding and subsequent amplification.

Nowadays, the antigen testing is widely used in the population due to its rapidity and simplicity in obtaining results. Some studies have evaluated the sensitivity of these tests compared to RT-qPCR, with sensitivities ranging from 58.26% to 81.1% in samples with Ct ≤ 30 [25,26]. The WHO has determined that the acceptable detection limit for an antigen test is 1000 genomic copies/μL with a sensitivity ≥ 80% and a specificity ≥ 97% in a 3-step (or less) procedure [21]. Our RT-LAMP test achieves 100% sensitivity and specificity with a limit of detection of Ct ≥ 30 (100–150 copies). Therefore, although the sensitivity is lower than RT-qPCR using samples without RNA extraction, our RT-LAMP test should be considered a useful diagnostic tool because patients with Ct > 30 are considered non-infective and have little ability to spread the virus [27]. In addition, the results obtained improve many RT-LAMP techniques developed by other groups. Over the past two years many SARS-CoV-2 RT-LAMP tests have been developed. In most of these cases, the samples were purified previously to amplification reaction [28,29,30,31,32,33], while in others, where the sample was not purified, a decrease in sensitivity was observed [19,34,35]. Several studies showed a detection limit of viral particles and a specificity like our test, but with a lower sensibility (85%) [36,37]. Lalli and colleagues used a Proteinase K (PK) based sample treatment, but we performed similar tests with minor modifications and obtained results that indicate a lack of PK ability to eliminate differences between samples (Figures S2 and S3).

Our test has been validated on a considerable number of negative and positive individuals with a wide range of viral loads obtained by RT-qPCR, and it was compared with other works that have used a small number of samples and some of them with very high viral loads (Cts between 20 and 24 in 10 out of 15 samples) [36,37,38]. In addition, many of the published procedures do not use an internal control to verify the correct performance of the whole ntire protocol, which is very important for clinical application, nor do they analyze the stability of the samples and reagents [36,37,38]. Finally, commercial kits have been tested with low sensitivity (77% for Ct ≤ 30) in agreement with other similar tests [39,40].

One of the limitations of this test is that it could not detect the new variants of SARS-CoV-2 that have emerged since 2019, especially if the mutations are in the region where the primers hybridize. This could be solved changing the target sequences of the specific primers for SARS-CoV-2 detection. In the same way, this test could be applied to other viral pathogens that require a rapid and simple diagnosis, simply by changing the target of the specific primers.

## 5. Conclusions

Our developed test would allow the detection of SARS-CoV-2 in a range of viral load values accepted by the WHO, improving on the currently most widely used technique at present (antigens), and being simpler than RT-qPCR, with high sensitivity and specificity, and including an internal control to reduce false negative results. Therefore, it could be used in many settings, including resource-limited areas, where vaccination coverage is lower and where there is no specific treatment or therapy. This technique could also be used for population screening as it can detect the presence/absence of SARS-CoV-2 in only 40 min using colorimetric detection and 30 min using fluorescence detection, if dedicated equipment is available.

## Figures and Tables

**Table 1:** Sequences of primers and probes for *N*, *RdRP* and *LYSOZYME* genes for RT-qPCR.

Gene	Primer and Probe Sequence (5′ → 3′)	
*N*	Forward	TTACAAACATTGGCCGCAAA
	Reverse	GCGCGACATTCCGAAGAA
	Probe	Cy5-ACAATTTGCCCCCAGCGCTTCAG-BHQ-3
*RdRP*	Forward	GGCCTCACTTGTTCTTGCTC
	Reverse	GCCACACATGACCATTTCAC
	Probe	HEX-CGTGTTGTAGCTTGTCACACCGTTTC-BHQ-2
*LYSOZYME*	Forward	CGCTACTGGTGTAATGATGGC
	Reverse	ACGGACAACCCTCTTTGC
	Probe	Atto 425-GCCTGTCATTTATCCTGCAGTGCTTTGC-BHQ-1

**Table 2:** Sequences of primers for *N* and *GAPDH* genes for RT-LAMP.

Gene	Primer and Sequence (5′ → 3′)	
*N*	F3	CGGCAGTCAAGCCTCTTC
	B3	TTGCTCTCAAGCTGGTTCAA
	FIP	TCCCCTACTGCTGCCTGGAGCGTTCCTCATCACGTAGTCG
	BIP	TCTCCTGCTAGAATGGCTGGCATCTGTCAAGCAGCAGCAAAG
	LF	TGAATTTCTTGAACTGTTG
	LB	TGGCGGTGATGCTGCTCT
*GAPDH*	F3	TCCACCCATGGCAAATTCC
	B3	AATGAGCCCCAGCCTTCT
	FIP	AGGGATCTCGCTCCTGGAAGATTCAAGGCTGAGAACGGGA
	BIP	AATCAAGTGGGGCGATGCTGGCCATGGTGGTGAAGACGC
	LF	GATGGGATTTCCATTGATGAC
	LB	CTGAGTACGTCGTGGAGTCC

## Data Availability

The data that support the findings of this study are available from the corresponding author upon reasonable request.

## Supplementary Materials

The following supporting information can be downloaded at: https://www.mdpi.com/article/10.3390/biology12070900/s1, Table S1: Tested conditions of trypsin treatment, Figure S1: RT-LAMP amplification curve of 15 trypsin treated samples, Figure S2: Amplification curves of SARS-CoV-2 by fluorescent RT-LAMP of two PK treated positive nasopharyngeal samples diluted at several concentrations in 2 negative buccal samples, Figure S3: Sample treatment with Proteinase K.
