# Supplementary material for: Sample Treatment with Trypsin for RT-LAMP COVID-19 Diagnosis
Source: Biology (Basel). 2023 Jun 23;12(7):900. doi: 10.3390/biology12070900 (PMC10376771; doi:10.3390/biology12070900)
Supplement: Supplementary file 1 [file biology-12-00900-s001.zip › Supplementary Figure S1.pdf]

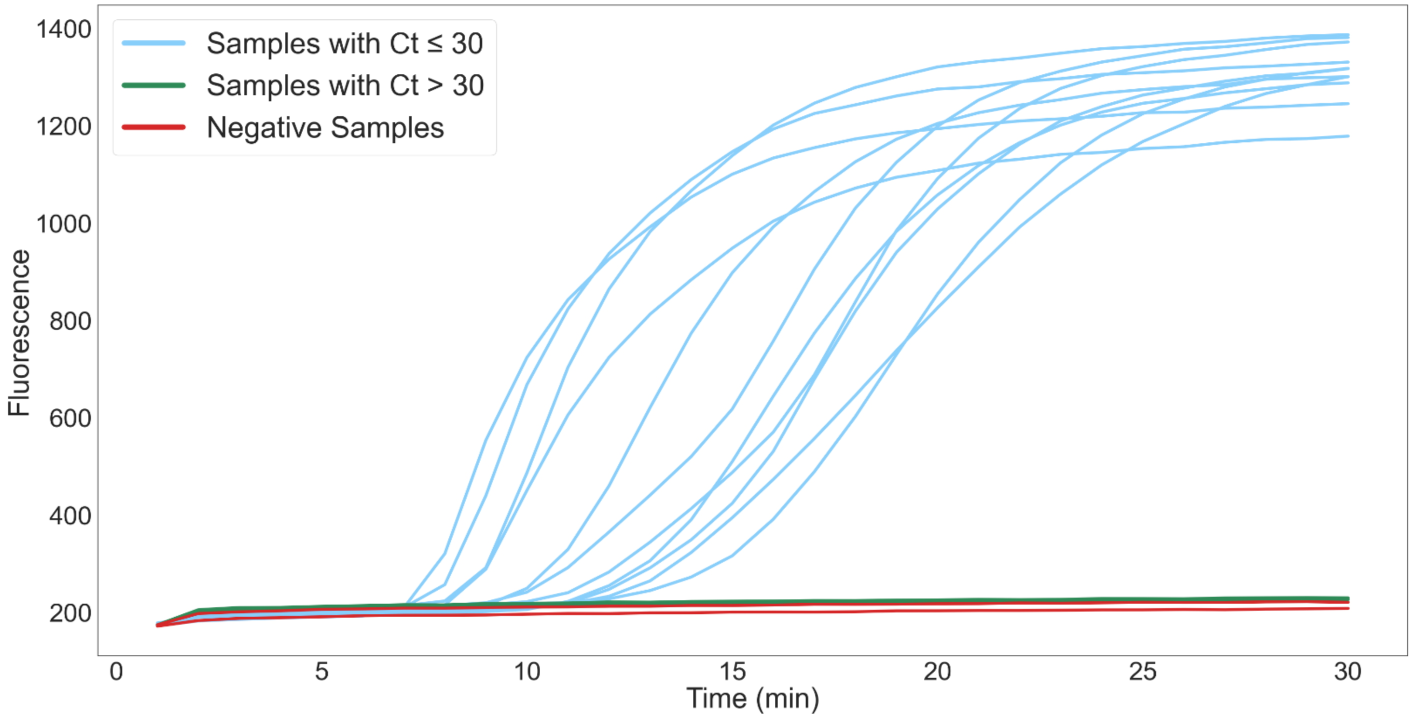

Supplementary Figure S1. RT-LAMP amplification curve of 15 trypsin treated samples. Eleven positive samples with a viral load above 100 copies (Ct ≤ 30), 2 samples with a viral load below 100 copies (Ct > 30) and 2 negative samples, quantified by RT-qPCR, were treated with trypsin, and amplified by fluorescent RT-LAMP. The graph shows how all samples with a viral load above 100 copies amplified in less than 13 min, except the 2 samples with the lowest viral load.
