# Supplementary material for: Sample Treatment with Trypsin for RT-LAMP COVID-19 Diagnosis
Source: Biology (Basel). 2023 Jun 23;12(7):900. doi: 10.3390/biology12070900 (PMC10376771; doi:10.3390/biology12070900)
Supplement: Supplementary file 1 [file biology-12-00900-s001.zip › Supplementary Figure S2.pdf]

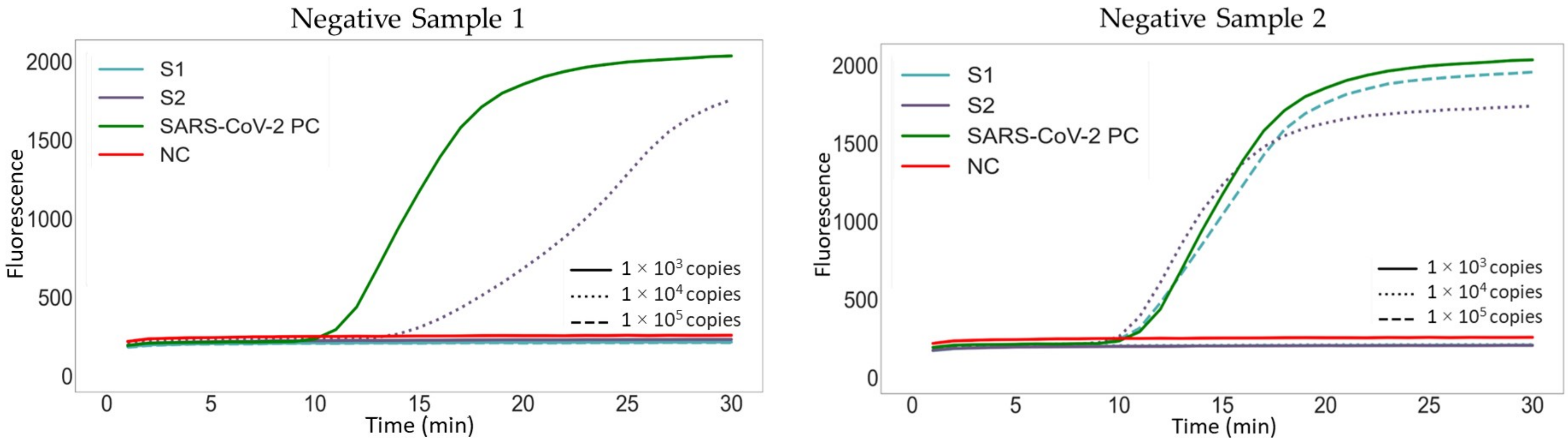

Supplementary Figure S2. PK treatment in several dilutions of positive SARS-CoV-2 samples in two negative buccal samples. Two positive nasopharyngeal samples (S1 and S2) quantified by RT-qPCR were diluted to 1×10^5, 1×10^4 and 1×10^3 copies and 1×10^4 and 1×10^3 copies respectively, in two negative buccal samples. Sample treatment with Proteinase K and fluorescent RT-LAMP reaction was performed in the ten dilutions. A lack of correlation between buccal samples treated with PK was observed: the same positive sample diluted in two different negative samples gave different results. PK treatment consisted of incubation of the sample at room temperature for 5 min at a PK ratio of 1/10 and inactivation at 95 °C for 5 min.. NC: negative PCR control performed with water. SARS-CoV-2 PC: synthetic SARS-CoV-2 RNA (1 x 103 copies). PK: Proteinase K (Thermo Fisher Scientific, ABgene, UK).
