# Supplementary material for: Sample Treatment with Trypsin for RT-LAMP COVID-19 Diagnosis
Source: Biology (Basel). 2023 Jun 23;12(7):900. doi: 10.3390/biology12070900 (PMC10376771; doi:10.3390/biology12070900)
Supplement: Supplementary file 1 [file biology-12-00900-s001.zip › Supplementary Figure S3.pdf]

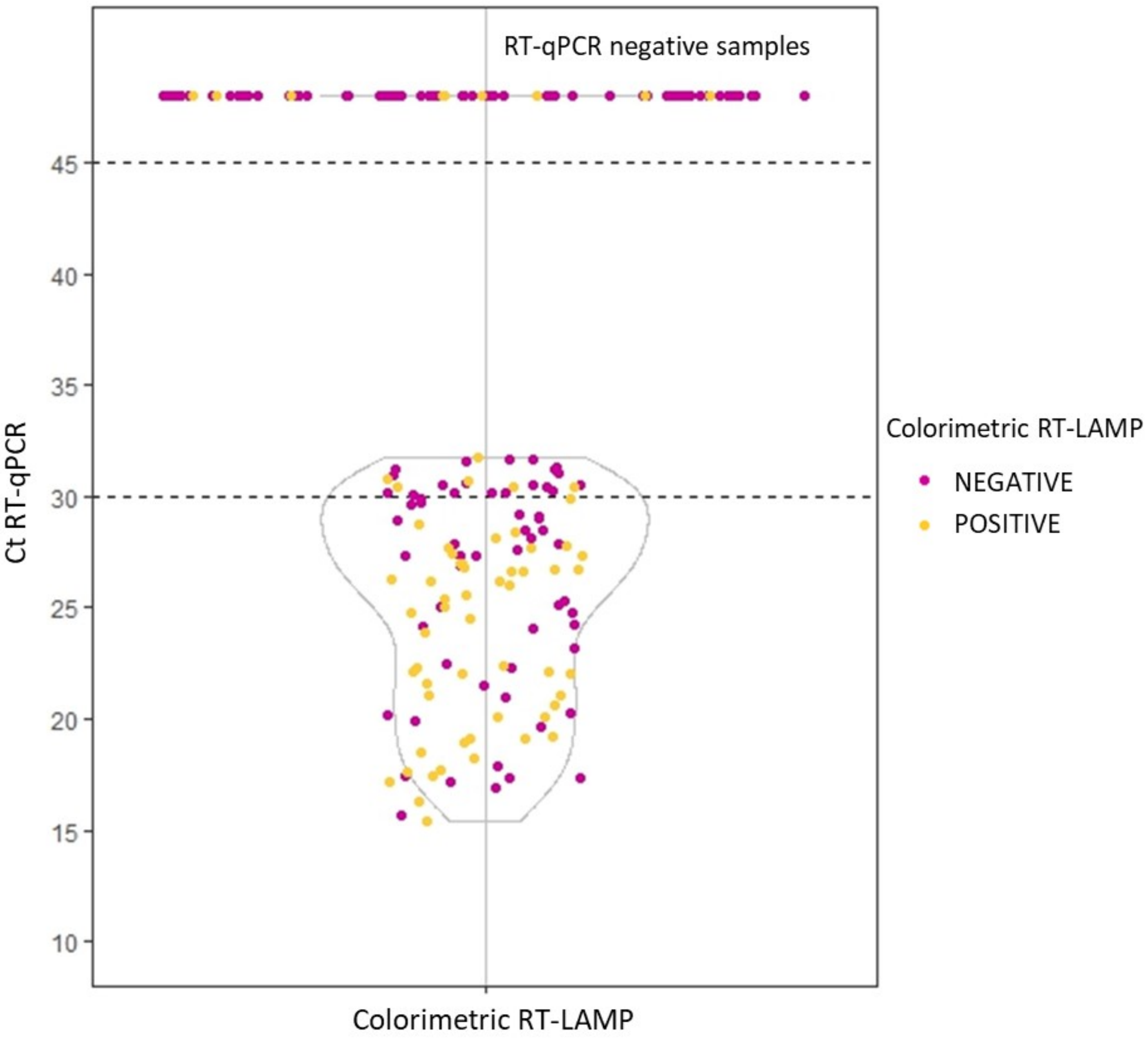


**Supplementary Figure S3.** Sample treatment with Proteinase K. Samples were incubated with PK ratio 1/10 5 min at room temperature and inactivated for 5 min at 95 °C. The violin plot shows the results of the colorimetric RT-LAMP validation on 195 PK-treated samples. Colorimetric RT-LAMP results were compared with RT-qPCR of extracted samples (non PK-treated) and sensitivity and specificity achieved was 86.15% and 54.62%, respectively. (PK: proteinase K, Thermo Fisher Scientific, ABgene, UK).
