# Supplementary material for: Sample Treatment with Trypsin for RT-LAMP COVID-19 Diagnosis
Source: Biology (Basel). 2023 Jun 23;12(7):900. doi: 10.3390/biology12070900 (PMC10376771; doi:10.3390/biology12070900)
Supplement: Supplementary file 1 [file biology-12-00900-s001.zip › Supplementary Table S1.pdf]

Supplementary Table S1. Trypsin treatment conditions tested.

Trypsin/Sample ratio	Incubation	Trypsin deactivation
1/10	10 min 37 °C	5 min 95 °C<br>5 min 65 °C<br>-
	5 min 37 °C	5 min 95 °C
1/3	10 min 37 °C	5 min 95 °C<br>5 min 65 °C
	10 min Room Temperature	5 min 95 °C<br>5 min 65 °C
	5 min Room Temperature	5 min 95 °C<br>5 min 65 °C
